# A novel upregulated LncRNA‐AC026150.8 promotes chemo‐resistance and predicts poor prognosis in acute myeloid leukemia

**DOI:** 10.1002/cam4.4349

**Published:** 2021-10-19

**Authors:** Henan Zhang, Yue Zhao, Xuan Liu, Yusi Liu, Xiaohui Wang, Yu Fu, Shuang Fu, Jihong Zhang

**Affiliations:** ^1^ Hematology Laboratory Shengjing Hospital of China Medical University Shenyang China

**Keywords:** acute myeloid leukemia, bioinformatics, cancer biology, LncRNA, prognosis

## Abstract

**Background:**

AML is a common hematological malignancy with poor prognosis, the pathogenesis is still unclear. lncRNA takes part in occurrence and development of AML. This research aims to explore new differentially expressed lncRNAs and their effects on AML.

**Methods:**

Database‐based bioinformatics analysis was performed to screen differentially expressed lncRNA in AML, real‐time PCR was used to analyze gene expression. Kaplan–Meier survival analysis was performed to determine prognostic effect of AC026150.8 in AML. The cell drug resistance experiment was performed to test effect of AC026150.8 on chemo‐resistance of AML cells. Catrapid online software and RNA pull‐down, mass spectrometry, western‐blot were used to predict and verify the combination of AC026150.8 and RNA splicing factors.

**Results:**

AC026150.8 was upregulated in AML patients and related to poor prognosis. High leukocyte counts, FAB classification, MLL‐AF9 expression and NPM1 mutations were associated with high AC026150.8 expression. Upregulated of AC026150.8 increased the drug resistance of AML cells. AC026150.8 could be combined with splicing factor PCBP1.

**Conclusions:**

For the first time, our study found that the upregulated AC026150.8 in AML is related to poor prognosis, overexpression of AC026150.8 could increase drug resistance of AML cells, and confirmed its scaffolding effect in combination with splicing factors. It is necessary to further study AC026150.8 and its downstream target genes to clarify the mechanism of AC026150.8 in AML.

## Graphical abstract

We found a new upregulated LncRNA‐AC026150.8 in AML patients, which was related to poor prognosis. The upregulated AC026150.8 increased the drug resistance of AML cells. AC026150.8 can interact with the splicing factor PCBP1 through scaffolding, and it is speculated that AC026150.8 can regulate the drug resistance of AML cells through this effect.

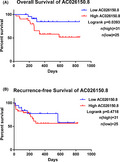

## 1 INTRODUCTION

Acute Myeloid Leukemia (AML) is a common, highly aggressive hematological tumor, characterized by abnormal proliferation of myeloid cells. In 2016, 85,000 patients died of AML worldwide, the number is anticipated to be doubled by 2040.^1^ Although the conventional chemotherapy and hematopoietic stem cell transplantation (HSCT) treatment strategies have made great progress in recent decades, due to the enhancement of drug resistance and high recurrence rate after chemotherapy, long‐term survival rate are not significantly improved.^2^, ^3^ At present, targeted drug development has also made some progress, but only three kinds of drugs (FLT3 inhibitors, IDH2 mutation inhibitors and KMT2A rearrangement inhibitors) targeted at coding gene abnormalities have been recommended by the 2017 European Leukemia Network (ELN), the international expert consensus on the diagnosis and management of adult AML and the National Comprehensive Cancer Network (NCCN) clinical practice guidelines,^4^, ^5^ therefore, it is urgent to explore molecular mechanisms of AML and new therapeutic targets to improve clinical prognosis of AML. In view of the high degree of heterogeneity of AML, about half of AML patients do not harbor protein‐coding genes or genetic abnormalities.^6^ Focusing on the abnormality of coding genes alone cannot fully clarify the mechanism of occurrence and development of AML. Therefore, we paid more attention to non‐coding RNA (ncRNA), which accounts for the vast majority (about 97%) of the human genome. Inhibitors of ncRNA (antisense oligonucleotides, double‐stranded RNA, interfering RNA) are easily obtainable and can quickly prevent or enhance the function of ncRNA, serving the purpose of treating tumors,^7^ thus seeking potential therapeutic targets among ncRNAs is a new approach in tumor therapeutic area.

Long noncoding RNA (lncRNA) is a type of ncRNA with over 200 nucleotides. The mechanisms of lncRNA mainly involve epigenetic/transcriptional regulation, chromatin modification, acting as sponges for miRNA, and as molecular scaffold recruiting proteins.^8^ Plenty of researches have shown that lncRNA is involved in the occurrence of AML, but current researches on its biological role in AML is mostly focused on the former three.^9^, ^10^, ^11^ It is rarely reported that lncRNA acts as molecular scaffold recruiting proteins in AML progression. The molecular scaffolding function of LncRNA refers to that lncRNA, as a structural component, brings proteins of same function close to each other through the scaffolding action to form a nucleic acid‐protein complex, thereby to carry out biological functions more efficiently. It is well known that proteins can function as molecular scaffolds.^12^ In contrast, RNA exhibits more advantages as scaffolds, for that RNA molecules do not require a translation step, and are capable of capturing multiple proteins at the same time^13^ and functionate immediately after transcription.^14^ In view of the wide existence of lncRNAs and the fast‐acting scaffolding characteristics of lncRNAs, exploring the scaffolding functions of lncRNAs is particularly important for studying the molecular mechanisms of disease occurrence and development. The molecular scaffold role of LncRNA has been investigated in non‐hematological tumors. NEAT1, GCAWKR, HOXA11‐AS, and other lncRNAs can act as molecular scaffolds to recruit histone methyltransferases and chromatin regulatory factors, forming complexes to regulate tumor biology process.^15^, ^16^, ^17^ However, lncRNA as a molecular scaffold is rarely reported in AML.

In this study, we dug out a novel differentially regulated lncRNA‐AC026150.8 in AML through database based bioinformation analysis. AC026150.8 was located on Chromosome 15:30,540,093–30,545,969 and Ensembl Gene ID of that is ENSG00000260693. AC026150.8 has not been studied in any tumor in the past. We will explore the expression, prognostic impact, drug resistance and the scaffolding function of AC026150.8 in AML.

## 2 METHODS

### 2.1 Data collecting and processing

A dataset of 173 AML samples from the TCGA database, along with clinical survival data, and a dataset of 337 normal samples from the GTEx database were obtained from the Xena Functional Genomics Explorer (https://xenabrowser.net/). AML‐M3 samples were excluded. Samples with incomplete clinical information were eliminated. Totally, 138 cases of AML were collected from TCGA database. The raw data was transformed to Exp‐count format. The EdgeR package (DESeq2) in R Bioconductor was applied to analyze the data on a local computer for differentially expressed genes (DEGs). DEGs was annotated with GENECODE v23 version. The significant difference was defined as: |log2 fold change|(FC) > 2 and adjusted *p*‐value (*p*‐adj) ≤ 0.01. Then, univariate cox analysis was applied to test the relationship between gene expression and prognostic risk with *p* < 0.05 as the significance threshold. Beta > 0 (HR > 1) indicated a poor prognosis. Gene Expression Profiling Interactive Analysis (GEPIA) online software based on the Cancer Genome Atlas (TCGA), was applied to further verify our analysis.

### 2.2 Clinical samples

One hundred and three bone marrow samples from AML patients and 18 samples from healthy donors were enrolled between January 2016 and July 2020. The diagnosis of AML was made according to French American British (FAB) and 2019 World Health Organization (WHO) criteria. The clinical data including age, gender, white blood cell (WBC), risk stratification and French‐American‐British (FAB) classification was collected. Patients’ risk stratification were based on the NCCN Guidelines version 1.2021 Acute Myeloid Leukemia (age ≥ 18 years). All patients included were newly diagnosed and did not receive any treatment before sampling. This study was reviewed and approved by the Medical Ethics Committee of Shengjing Hospital of China Medical University. All samples used in our study were clinical waste samples after testing, and the clinical information of the patients is obtained from the electronic medical record. Application for exemption of informed consent has been approved by the ethics committee.

### 2.3 Cell culture

AML cell line KG‐1 and K562 cells were purchased from Procell Life Science & Technology Co., Ltd. KG‐1 and K562 cells were respectively cultured in Iscove's Modified Dulbecco's Medium (IMDM) medium (Gibco) and Roswell Park Memorial Institute (RPMI) 1640 medium (Gibco), with 10% fetal bovine serum (FBS, Hyclone), 100 U penicillin and 100 mg/ml streptomycin (Gibco@) at 37°C in a humidified atmosphere of 5% CO_2_.

### 2.4 Overexpression vector construction, siRNA synthesis, and infection

For AC026150.8 overexpression, the sequence was synthesized and subcloned into pcDNA3.1 (pc‐AC026150.8). The empty plasmid pcDNA3.1 was used as control. Specific small interfering RNA (siRNA) targeting AC026150.8 mRNA (si‐AC026150.8, Sense 5′‐ACAAGGUGGUGGAGACAUUTT‐3′, Antisense 5′‐AAUGUCUCCACCACCUUGUTT‐3′) and negative control (si‐NC, Sense 5′‐UUCUUCGAACGUGUCACGUTT‐3′, Antisense 5′‐ACGUGACACGUUCGGAGAATT‐3′) were synthesized by Sangon. Cell transfection was performed using Lipofectamine 3000 (Invitrogen) according to the manufacturer's protocol. After 48 h (AC026150.8 overexpression) or 24 h (AC026150.8 knockdown), the incubation media were removed and the cells were harvested for further experiments.

### 2.5 RNA extraction and real‐time PCR

Total mRNA was extracted with TRIZOL (Invitrogen). Complementary DNA (cDNA) was synthesized using a PrimeScript™ RT reagent kit (TAKARA) with mRNA as the template. Real‐time PCR was performed on an ABI Prism 7500 detection system (Applied Biosystems) by using TAKARA SYBR^®^ Premix Ex Taq™ II kits (TAKARA) in a 20 μl reaction (2 μl of template cDNA, 2 μl of 5 μmol/L each primer, 10 μl of 2× SYBR Green Master Mix, 0.4 μl of ROXII, and 5.6 μl of ddH_2_O). Primer sequences for AC026150.8 were Forward: 5′‐CAGTCTCACCTTCCAGCGA‐3′ and Reverse: 5′‐ACCAGTAGTCAGGACGGCTC‐3′; the internal reference gene was glyceraldehyde 3‐phosphate dehydrogenase (GAPDH), and the primer sequences were Forward: 5′‐GAAGGTCGGAGTCAACGGAT‐3′ and Reverse: 5′‐CCTGGAAGATGGTGATGGGAT‐3′. The PCR cycling parameters were as following: predenaturation at 95°C for 30 s, 45 cycles of denaturation at 95°C for 5 s, and annealing at 60°C for 20 s. Relative levels of AC026150.8 were calculated according to the ΔΔCt method.^18^

### 2.6 IC50 and cytotoxicity analysis

To detect the cytosine arabinoside (Ara‐C) resistance in KG‐1 and K562 Cells, cells were seeded in 96‐well plate, divided into six groups, with five wells in each group and 100 μl medium per well containing 5 × 10^3^ cells. Ara‐C was added immediately after cell inoculation. The final concentrations of Ara‐C for KG‐1 cell were 0, 0.625, 1.25, 2.5, 5, 7.5 μmol/L, and the final concentrations of Ara‐C for K562 cell were 2.5, 5, 10, 20, 45, 90, 180 μmol/L. After 48 h, CCK‐8 detected cell viability. Cell growth inhibition rate was determined as follows: (control group absorbance − experimental group absorbance)/(control group absorbance − blank group absorbance) × 100%. The median inhibitory concentration (IC50) of Ara‐C was calculated by SPSS software. In order to detect the effect of AC026150.8 on cytotoxicity, the experiment was divided into six groups (cell group, cell + Ara‐C group, negative control group, negative control + Ara‐C group, overexpression or knockdown AC026150.8 group, over‐expression or knock‐down AC026150.8 + Ara‐C group). After transfected with pc‐AC026150.8 for 48 h or with si‐AC026150.8 for 24 h, KG‐1 or K562 cells were received Ara‐C treatment at concentration of their respective IC50, and then, for 48 h later, cell viability was detected.

### 2.7 Identification of AC026150.8 binding proteins

The sequence of AC026150.8 was obtained from UCSC Genome database (http://genome.ucsc.edu/) and put into catRAPID omics tools (http://service.tartaglialab.com/) for binding protein prediction,^19^ then, predicted protein and binding sites of protein/RNA were downloaded.

### 2.8 RNA pull‐down assays

Pierce Magnetic RNA‐Protein Pull‐Down Kit (Thermo Fisher) was used for RNA pull‐down assays. In vitro transcribed (IVT) RNA probes for pull‐down assays were prepared with AmpliScribe™ T7 High Yield Transcription Kit (Epicentre). In brief, 1 × 10^7^ cells were collected and washed in cold phosphate‐buffered saline. The cell pellets lysed in 1ml IP lysis buffer, and centrifuged at 12,000 *g* for 15 min at 4°C to collect the supernatant. Second, 50 μl washed streptavidin magnetic beads incubated with 5 μg biotinylated IVT lncRNA or its antisense RNA for 30 min at room temperature with agitation. Then, probes coated beads incubated with 500 μl cell lysis supernatant for 1 h. The beads were washed briefly with wash buffer for five times and elutioned. The bound protein to the RNA were analyzed by mass spectrometry.

### 2.9 GO and pathway analysis

To analyze functions and pathways of the proteins interacting with AC026150.8, we performed Gene Ontology (GO) and Pathway analysis with David database (https://david.ncifcrf.gov/tools.jsp). The *p*‐value denotes the significance of GO/Pathway terms enrichment in the genes. The lower the *p*‐value, the more significant the GO/Pathway Term was. Terms containing 10 or more genes with a *p*‐value <0.05 was considered interested terms.

### 2.10 Western‐blot analysis

After quantification by the BCA method, the bound protein to the RNA was electrophoresed on a 10% SDS‐polyacrylamide gel, and then transferred to a polyvinylidene fluoride (PVDF) membrane (Millipore). The membrane was blocked with 5% skimmed milk in PBST containing 0.1% Tween‐20 for 60 min and combined with the main rabbit anti‐FUS or PCBP1 Antibody (FUS, abs137868, 1:1000, Absin; PCBP1, 14523‐1‐AP, 1:1000, Proteitech) and mouse anti‐GAPDH Antibody (sc‐365062, 1:10,000, Santa Cruz Biotechnology) overnight at 4°C, and then combined with secondary polyclonal goat anti‐rabbit/mouse HRP‐conjugated antibody (ZDR‐5306/5307, 1:2500, ZSGB‐bio) for 2 h at room temperature. Use the enhanced function to visualize the signal Chemiluminescence (ECL, Millipore) reagents. GAPDH was used as a loading control.

### 2.11 Statistical analysis

All statistical analyses were performed with GraphPad Prism 6.0 (GraphPad software) and data were presented as mean ± standard error. We repeated all experiments at least three times. Differences between groups were analyzed via Student's *t* test and differences among three or more groups were analyzed via one‐way analysis of variance (ANOVA) followed by Bonferroni's multiple comparison. Non‐parametric test was used to compare the differences in groups with unequal variances. Survival analysis was performed using Kaplan–Meier analysis. The association between AC026150.8 expression and age/gender and WBC count were analyzed by Pearson's chi‐square test. The relation between AC026150.8 expression and French–American–British (FAB) category and risk stratification were analyzed via likelihood ratio chi‐square test. *p* < 0.05 was considered statistically significant. *p < 0.05, **p < 0.01, ***p < 0.001.

## 3 RESULTS

### 3.1 Screening the different LncRNA in AML

To identify potential lncRNA biomarkers, we compared the AML patients in TCGA cohort with normal samples from the GTEx cohort. A total of 60,498 genes were obtained. After filtered by absolute FC > 2 and *p*‐adj ≤ 0.01, 12,027 DEGs were screened out, of which 3038 genes were lncRNA, including 1187 LincRNAs. By using univariate cox analysis, 152 LincRNAs were identified as prognosis associated differentially expressed lncRNAs (*p* < 0.05), among them, 36 LincRNAs were Upregulated (FC > 2) and poor prognosis (Beta >0 or HR > 1) (File S1). After taking intersection with GEPIA online software (http://gepia.cancer‐pku.cn/), only two up‐regulated (FC > 2) with poor prognosis LincRNAs were left, KIAA0125 and AC026150.8. AC026150.8 was a new lncRNA locating on chromosome 15:30,540,093–30,545,969 that had never been reported yet. The Ensembl Gene ID of AC026150.8 was ENSG00000260693, and the Transcript ID of that was ENST00000562992.1.

### 3.2 Analysis of AC026150.8 expression and prognosis with Gepia software

**FIGURE 1:**
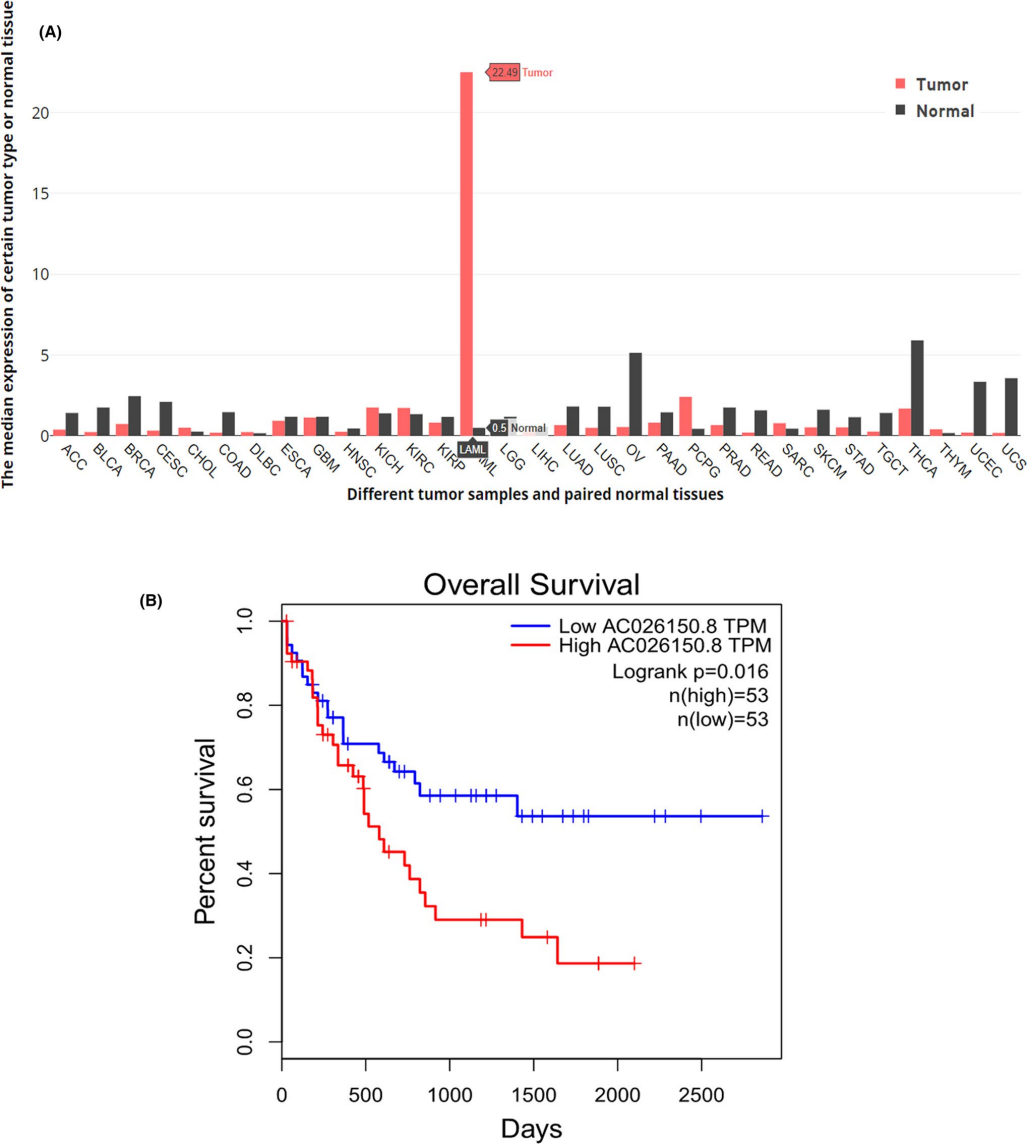
AC026150.8 expression and prognosis from gepia online sofeware. (A) The gene expression profile across all tumor samples and paired normal tissues. (B) Overall survival (OS) in group of patients with differential expression of AC026150.8. ACC, Adrenocortical carcinoma; BLCA, Bladder Urothelial Carcinoma; BRCA, Breast invasive carcinoma; CESC, Cervical squamous cell carcinoma and endocervical adenocarcinoma; CHOL, Cholangio carcinoma; COAD, Colon adenocarcinoma; DLBC, Lymphoid Neoplasm Diffuse Large B‐cell Lymphoma; ESCA, Esophageal carcinoma; GBM, Glioblastoma multiforme; HNSC, Head and Neck squamous cell carcinoma; KICH, Kidney Chromophobe; KIRC, Kidney renal clear cell carcinoma; KIRP, Kidney renal papillary cell carcinoma; LAML, Acute Myeloid Leukemia; LGG, Brain Lower Grade Glioma; LIHC, Liver hepatocellular carcinoma; LUAD, Lung adenocarcinoma; LUSC, Lung squamous cell carcinoma; MESO, Mesothelioma; OV, Ovarian serous cystadenocarcinoma; PAAD, Pancreatic adenocarcinoma; PCPG, Pheochromocytoma and Paraganglioma; PRAD, Prostate adenocarcinoma; READ, Rectum adenocarcinoma; SARC, Sarcoma; SKCM, Skin Cutaneous Melanoma; STAD, Stomach adenocarcinoma; TGCT, Testicular Germ Cell Tumors; THCA, Thyroid carcinoma; THYM, Thymoma; UCEC, Uterine Corpus Endometrial Carcinoma; UCS, Uterine Carcinosarcoma

Gepia online software was used to predict the expression and prognosis of AC026150.8 in AML. AC026150.8 was dramatically increased by proximately 45 times in the AML group comparing with the normal group. AC026150.8 was also increased in Kidney Chromophobe, Kidney renal clear cell carcinoma and Pheochromocytoma and Paraganglioma. But in other cancers included in Gepia, the expression of AC026150.8 is lower than in the normal group (Figure 1A).

Overall survival (OS) analysis was performed by Survival Plots Analysis on GEPIA website based on gene expression. The high AC026150.8 group had a shorter OS (median, *p* < 0.05; Figure 1B).

### 3.3 Correlation analysis between clinicopathological characteristics of AML patients and AC026150.8 expression

**FIGURE 2:**
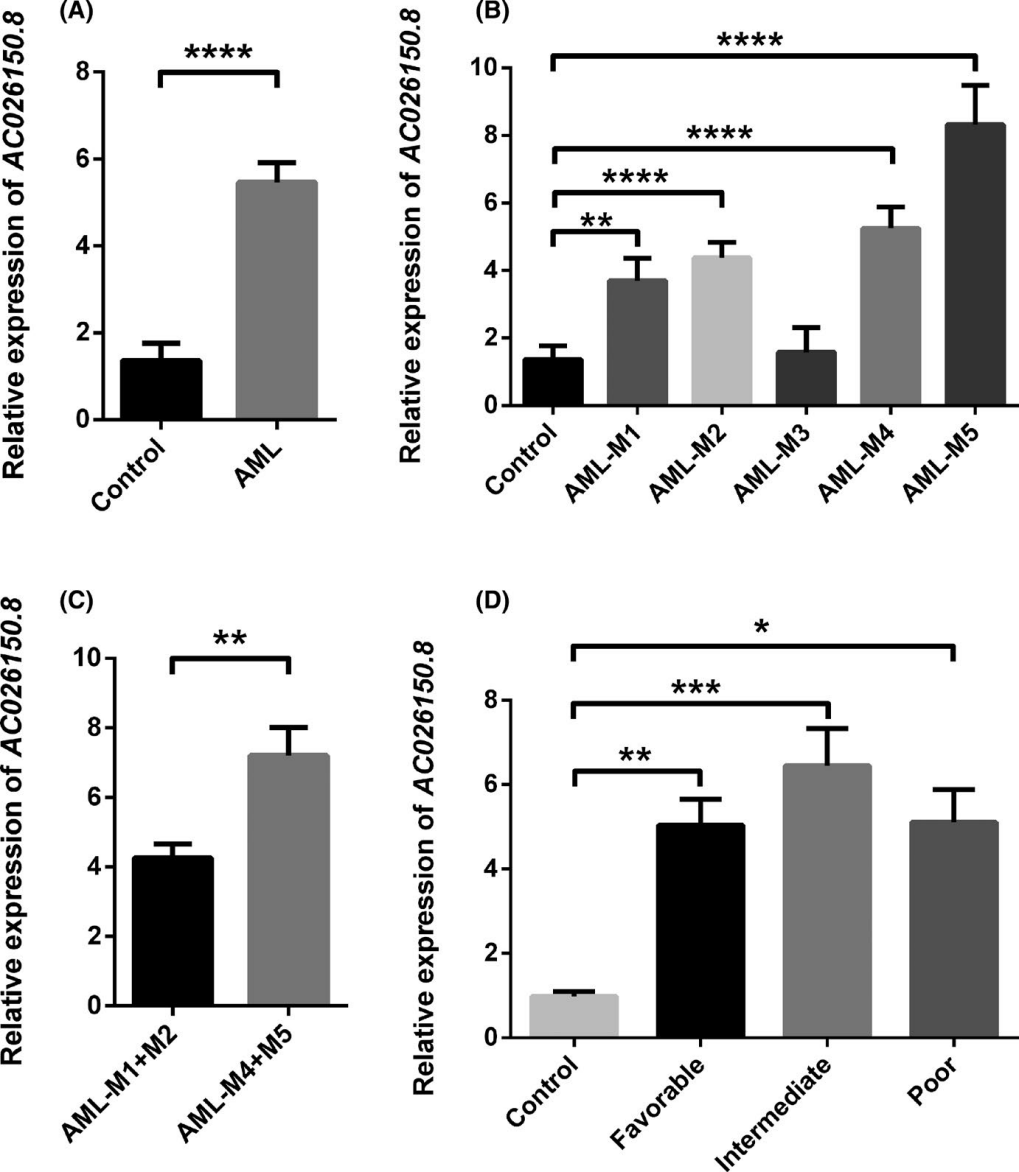
AC026150.8 expression was measured by Real‐time PCR. (A) Comparison of AC026150.8 expression between AML patients and normal controls. (B) Comparison of AC026150.8 expression between AML patients with different FAB classification and normal controls. (C) AC026150.8 expression of M4 and M5 patients were significantly higher than that of M1 and M2 patients. (D) Comparison of AC026150.8 expression among the different risk stratification groups. *p < 0.05, **p < 0.01, ***p < 0.001, ****p < 0.0001

Gene expression levels of AC026150.8 were detected with real‐time quantitative PCR on bone marrow tissues from 103 AML patients and 18 healthy donors. Our results showed that AC026150.8 was upregulated in AML patients compared to normal controls (Figure 2A). According to FAB criteria, AML patients were divided to M1, M2, M3, M4 and M5. AC026150.8 was significantly increased in M1, M2, M4 and M5,when compared with normal controls (*p* values were 0.0043, <0.0001, <0.0001, <0.0001, respectively), but no significant difference was observed between M3 and normal controls (*p* > 0.05) (Figure 2B). In order to explore whether the expression of AC026150.8 is related to abnormal monocyte development, the AC026150.8 expression was compared between M1 and M2 patients and M4 and M5 patients, and the result showed that the AC026150.8 expression of M4 and M5 patients was significantly higher than that of M1 and M2 patients (*p* = 0.0015; Figure 2C).

According to NCCN guidelines of AML, risk stratification was classified as favorable, intermediate, and poor based on cytogenetics and molecular genetics of AML patients. To clarify the relationship between AC026150.8 expression and risk stratification, the AC026150.8 expression was evaluated among patients with different risk stratification. AC026150.8 was highly expressed in all the three risk groups (*p* < 0.05), but no significant difference was found among these different risk groups (*p* > 0.05; Figure 2D).

**FIGURE 3:**
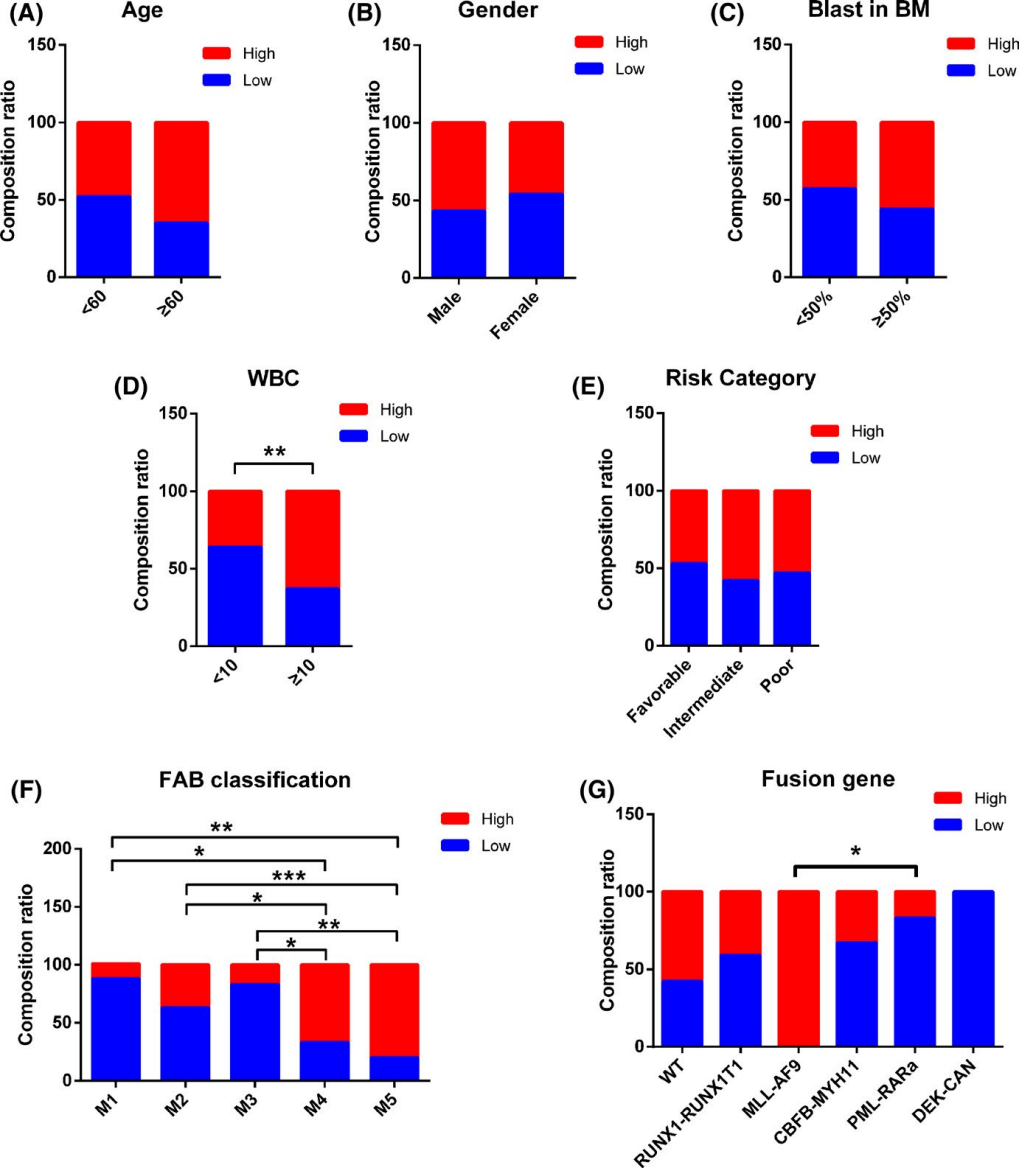
Percentage of patients with differential expression of AC026150.8 grouped by age (A), gender (B), blast ratio (C), WBC level (D), risk category (E), FAB calssification (F) and fusion gene (G). High leukocyte counts, FAB classification M4 and M5, MLL‐AF9 expression were associated with high expression of AC026150.8. *p < 0.05, **p < 0.01, ***p < 0.001

**FIGURE 4:**
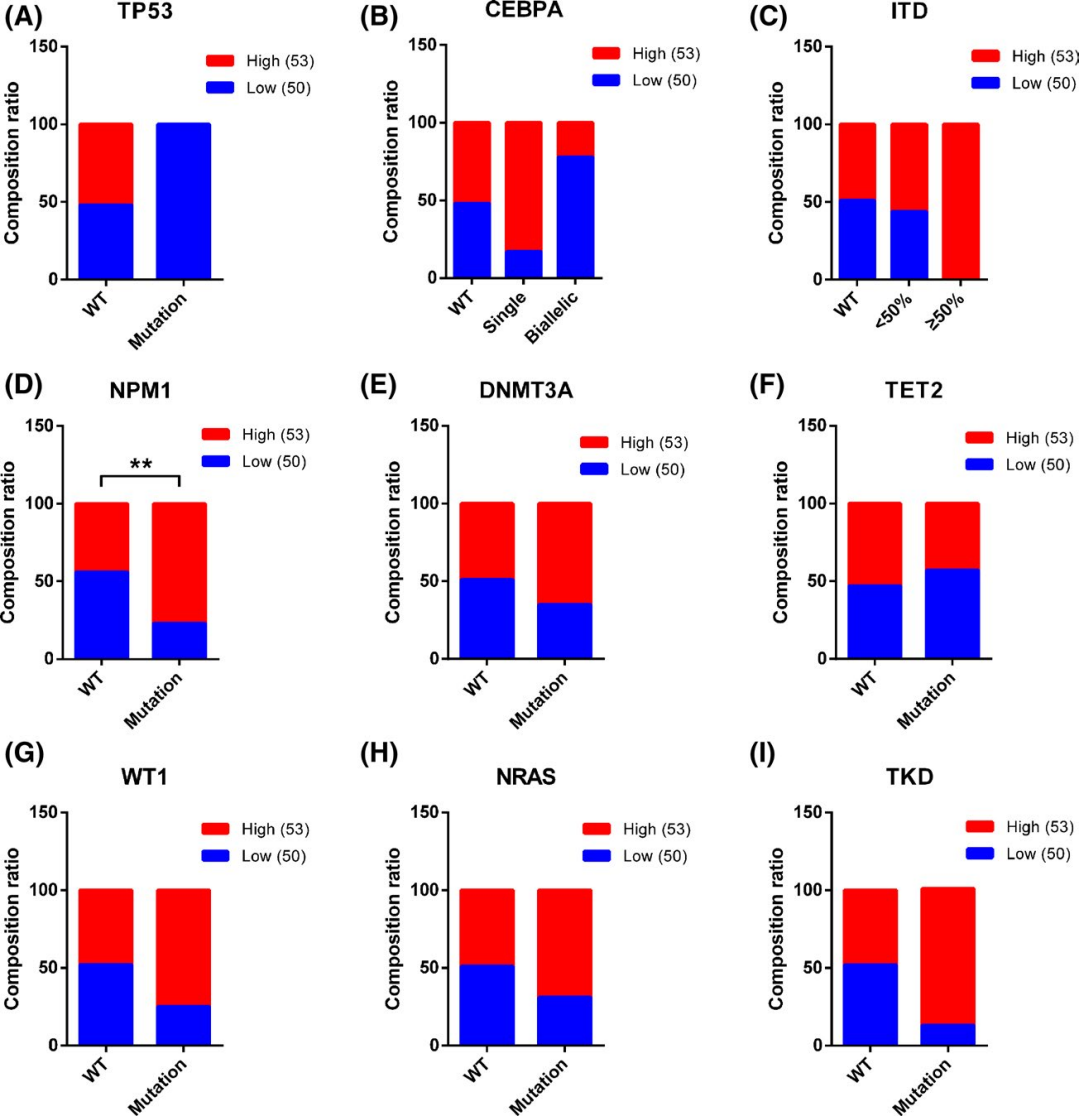
Percentage of patients with differential expression of AC026150.8 grouped by accompanied gene mutation TP53(A), CEBPA(B), ITD(C), NPM1(D), DNMT3A(E), TET2(F), WT1(G), NRAS(H) and TKD(I). NPM1 mutation was associated with high expression of AC026150.8. **p < 0.01

To investigate the correlation between AC026150.8 expression and clinical characteristics, AML patients were divided into low AC026150.8 group (*n* = 50, fold‐change > median) and high AC026150.8 group (*n* = 53, fold‐change ≤ median). Our result demonstrated that most newly diagnosed patients with high white blood cell (WBC) count were more likely to show high expression of AC026150.8 (*p* = 0.0081) (Table 1; Figure 3D). Moreover, AC026150.8 expression was strongly correlated with FAB classification (*p* < 0.0001). Compared with patients with M1, M2, and M3, high AC026150.8 expression was more frequently observed in M4 and M5 patients (Tables 1 and 2; Figure 3F). AC026150.8 expression was also associated with fusion gene (*p* = 0.0419). Compared with PML‐RARa positive patients, high expression of AC026150.8 was more often observed in patients with MLL‐AF9 (Tables 1 and 3; Figure 3G). However, the expression of AC026150.8 was not related to age (*p* = 0.134), gender (*p* = 0.282), the number of blast cells in the bone marrow (*p* = 0.212) or risk category (*p* = 0.652) in AML patients (Table 1; Figure 3A–C,E). To explore the relationship between AC026150.8 expression and gene mutation, we analyzed the correlation between common gene mutations in AML and AC026150.8 expression (Figure 4). Our result showed that NPM1 was associated with upregulated AC026150.8 (Figure 4D).

**TABLE 1:** Correlation analysis between clinicopathological characteristics of AML patients and AC026150.8 expression

Parameters	*n*	AC026150.8 expression		OR	95% CI	*p*‐Value
		Low (50)	High (53)			
Age (years)		50	53			
<60	80	42	38	2.072	0.7903–5.434	0.1341
≥60	23	8	15			
Gender						
Male	53	23	30	0.653	0.3001–1.421	0.2819
Female	50	27	23			
WBC (×10^9/L)						
<10	44	28	16	2.943	1.309–6.616	0.0081
≥10	59	22	37			
Blast in BM						
<50%		21	16	1.675	0.7433–3.772	0.2118
≥50%		29	37			
FAB classification						
M1	8	7	1	—	—	<0.0001
M2	41	26	15			
M3	6	5	1			
M4	18	6	12			
M5	30	6	24			
M6/M7	0	0	0			
Fusion gene						
WT	66	28	38	—	—	0.0419
RUNX1‐RUNX1T1	22	13	9			
MLL‐AF9	3	0	3			
CBFB‐MYH11	3	2	1			
PML‐RARa	6	5	1			
DEK‐CAN	1	1	0			
Risk category						
Favorable	57	30	27	—	—	0.6523
Intermediate	30	13	18			
Poor	16	7	8			
TP53 mutation						
WT	101	48	53	0.475	0.3872–0.5834	0.2332
Mutation	2	2	0			
CEBPA mutation						
WT	88	42	46	—	—	0.0625
Single allelic mutation	6	1	5			
Biallelic mutation	9	7	2			
ITD mutation						
WT	82	42	40	—	—	0.1393
<50%	18	8	10			
≥50%	3	0	3			
NPM1 mutation						
WT	81	45	36	2.444	1.104–5.413	0.0063
Mutation	22	5	17			
DNMT3A mutation						
WT	86	44	42	1.450	0.7373–2.850	0.2316
Mutation	17	6	11			
TET2 mutation						
WT	89	42	47	0.826	0.4988–1.367	0.4886
Mutation	14	8	6			
WT1 mutation						
WT	91	47	44	2.066	0.7599–5.617	0.153
Mutation	12	3	9			
NRAS mutation						
WT	90	46	44	1.661	0.7169–3.849	0.2824
Mutation	13	4	9			
TKD mutation						
WT	95	49	46	4.126	0.6526–26.090	0.0791
Mutation	8	1	7			

**TABLE 2:** Correlation analysis between FAB classification and 0.8 expression

Comparison of two groups	OR	95% CI	*p*‐Value
M1 versus M2	4.038	0.452–36.068	0.359
M1 versus M3	1.400	0.070–28.120	1.000
M1 versus M4	14.000	1.385–141.485	0.030*
M1 versus M5	28.000	2.869–273.276	0.001*
M2 versus M3	0.347	0.037–3.253	0.617
M2 versus M4	3.467	1.078–11.147	0.033*
M2 versus M5	6.933	2.314–20.774	<0.001***
M3 versus M4	10.000	0.944–105.921	0.061
M3 versus M5	20.000	1.954–204.728	0.006**
M4 versus M5	2.000	0.531–7.539	0.302

* *p* < 0.05

** *p* < 0.01

*** *p* < 0.001.

**TABLE 3:** Correlation analysis between fusion gene and AC026150.8 expression

Comparison of two groups	OR	95% CI	*p*‐Value
WT versus RUNX1‐RUNX1T1	0.51	0.1910–1.359	0.175
WT versus MLL‐AF9	1.079	0.99–1.176	0.266
WT versus CBFB‐MYH11	0.368	0.032–4.268	0.816
WT versus PML‐RARa	0.147	0.016–1.332	0.134
WT versus DEK‐CAN	0.966	0.901–1.034	0.433
RUNX1‐RUNX1T1 versus MLL‐AF9	1.333	0.962–1.848	0.096
RUNX1‐RUNX1T1 versus CBFB‐MYH11	0.722	0.057–9.217	1.000
RUNX1‐RUNX1T1 versus PML‐RARa	0.289	0.029–2.908	0.375
RUNX1‐RUNX1T1 versus DEK‐CAN	0.929	0.803–1.074	1.000
MLL‐AF9 versus CBFB‐MYH11	4	0.733–21.838	0.400
MLL‐AF9 versus PML‐RARa	4	0.733–21.838	0.048*
MLL‐AF9 versus DEK‐CAN	—	—	0.250
CBFB‐MYH11 versus PML‐RARa	0.4	0.016–10.017	1.000
CBFB‐MYH11 versus DEK‐CAN	0.667	0.300–1.484	1.000
PML‐RARa versus DEK‐CAN	0.833	0.583–1.192	1.000

* *p* < 0.05

### 3.4 Kaplan–Meier survival analysis for the prognosis of AC026150.8

**FIGURE 5:**
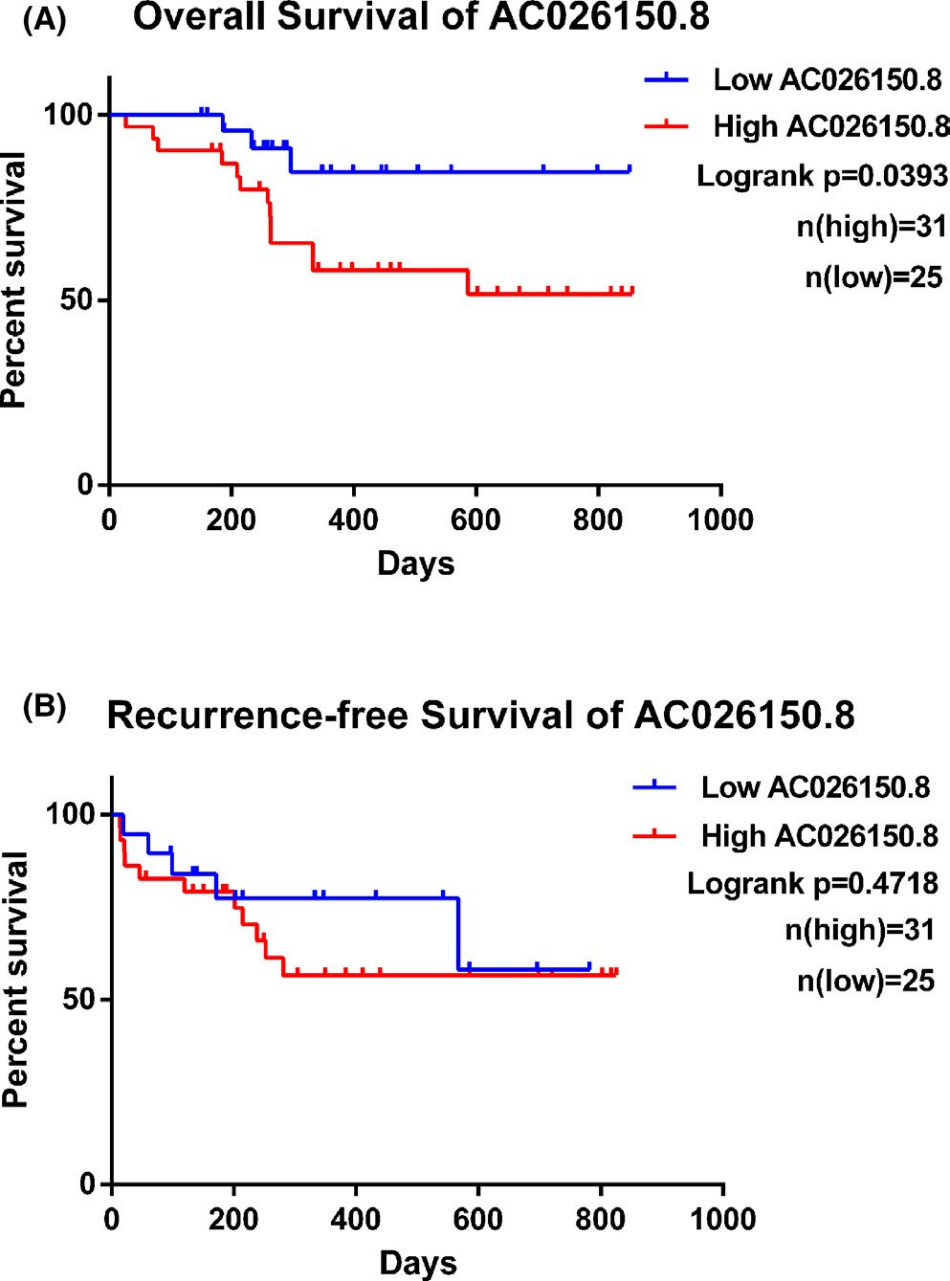
Kaplan–Meier analysis of OS and recurrence‐free survival (RFS) in group of patients with differential expression of AC026150.8. (A) OS of patients with differential expression of AC026150.8. (B) RFS of patients with differential expression of AC026150.8

All 56 patients received conventional chemotherapy for AML. Kaplan–Meier survival analyses results indicated that the high AC026150.8 group had a shorter OS (*p* = 0.0393; Figure 5A). The recurrence‐free survival of patients with high AC026150.8 trended to decrease, but the survival curve was not significantly different between the high and low AC026150.8 groups (*p* > 0.05; Figure 5B).

**FIGURE 6:**
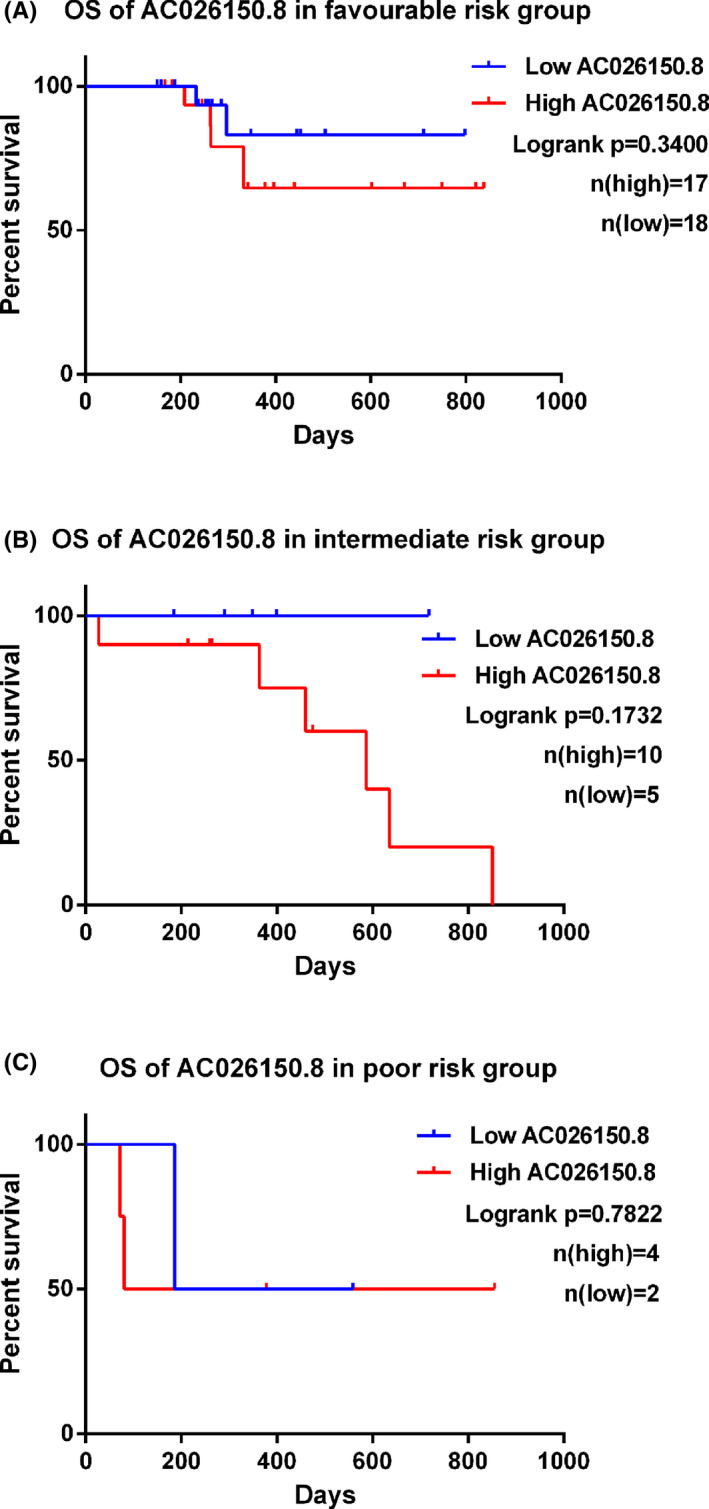
Kaplan–Meier analysis for OS on patients with differential expression of AC026150.8 in favorable risk group (A), intermediate risk group (B) and poor risk group (C), respectively

Then, Kaplan–Meier analysis of OS was also performed in patients with differential expression of AC026150.8 in different risk groups. The result showed that patients with high expression of AC026150.8 had a shorter OS in both favorable and intermediate risk groups (Figure 6A,B), but the difference was not statistically significant. In the poor risk group, no such trend was observed (Figure 6C). This may be due to too few samples in this group that can be followed up for prognosis. From the above, we speculated that high expression of AC026150.8 indicate a worse prognosis in patients with the same risk stratification, especially in favorable and intermediate risk groups.

### 3.5 Overexpression of AC026150.8 increased Ara‐C resistance in KG‐1 and K562 cells

**FIGURE 7:**
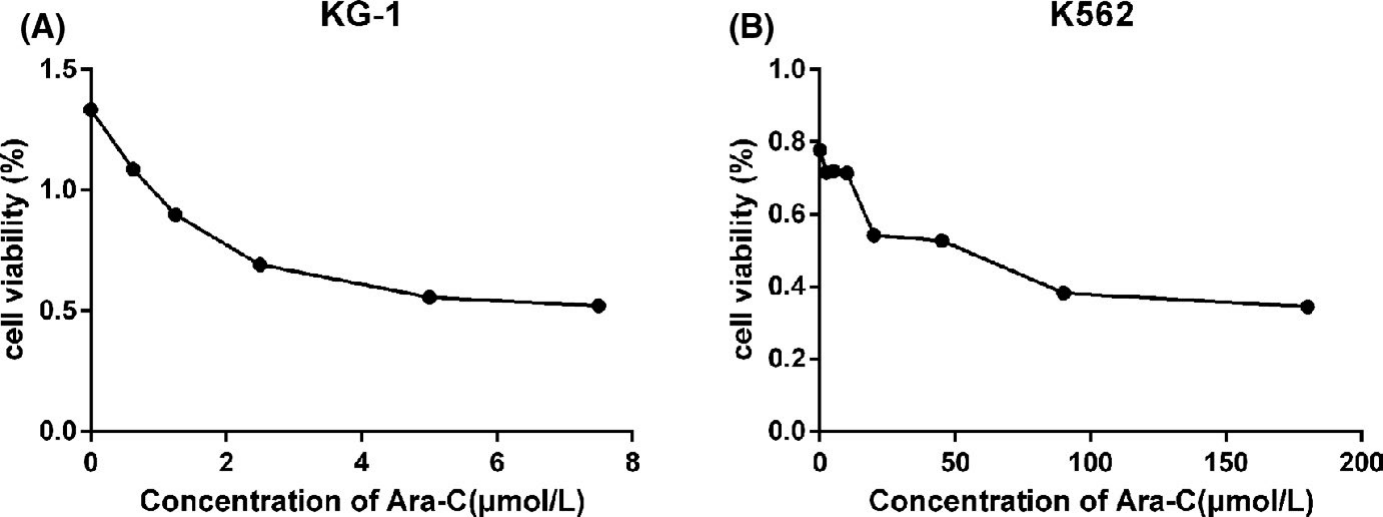
KG‐1 (A) and K562 (B) cell lines were cultured with increasing concentrations of Arc‐C for 48 h, and then cell viability was measured by CCK8 assay

**FIGURE 8:**
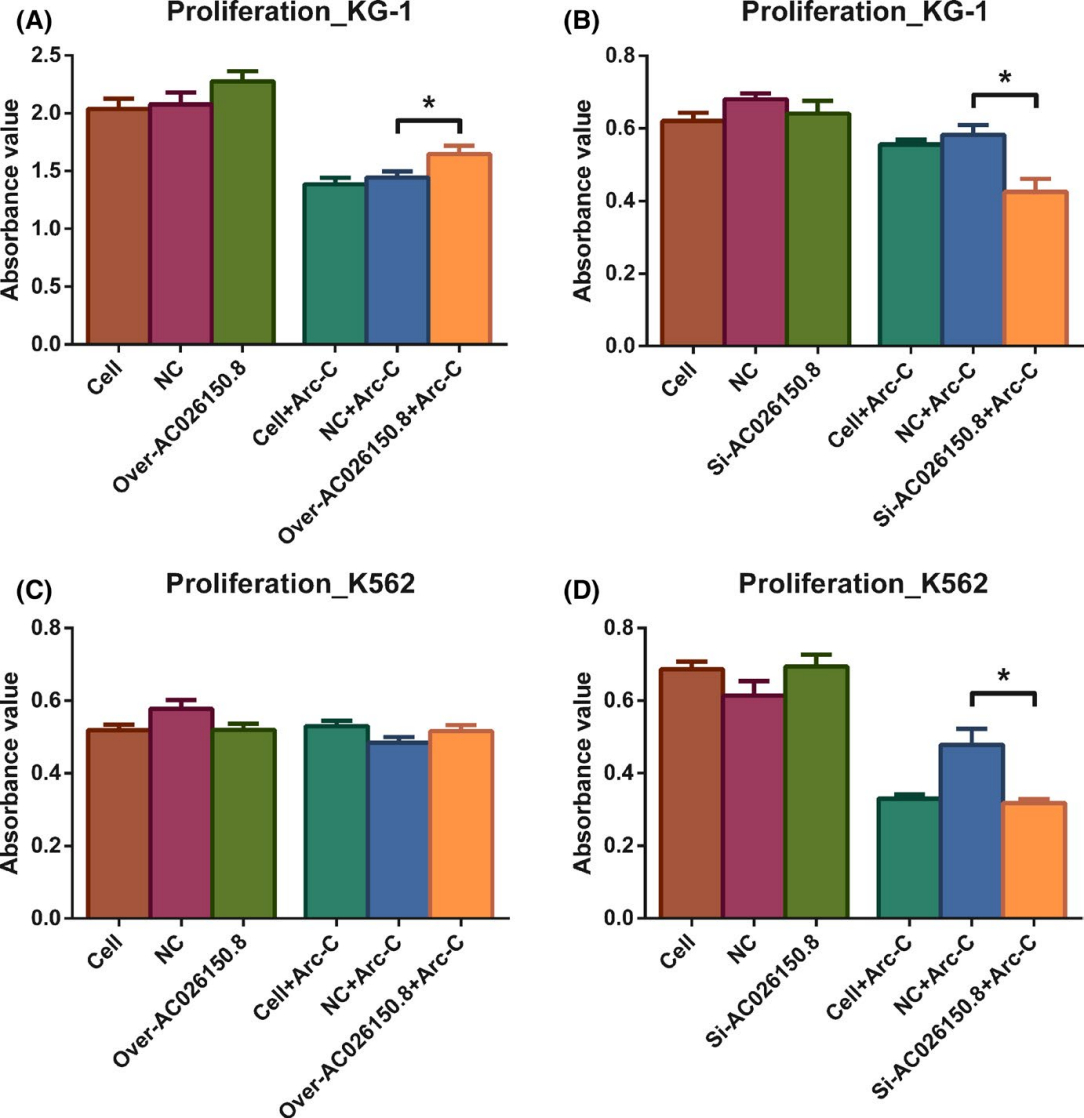
AC026150.8 promote cell drug resistance in KG‐1 and K562 cells. (A, B) In KG‐1 cells, overexpression or knockdown of AC026150.8 has no effect on cell activity, but affects the drug sensitivity of cell to Arc‐C. (C, D) In K562 cells, overexpression or knockdown of AC026150.8 has no effect on cell activity, but knockdown of AC026150.8 affects the drug sensitivity of cell to Arc‐C. *p < 0.05

The expression of AC026150.8 was significantly increased after pc‐AC026150.8 transfection. After treating with different increasing concentrations of Ara‐C to KG‐1 (0.625–7.5 μmol/L) and K562 (2.5–180 μmol/L) cells for 48 h, cell viability was measured by CCK8 assay, and the result showed that the inhibitory effect on cell viability was dose‐dependent (Figure 7). The IC50 of Ara‐C in KG‐1 cells was 3.13 μmol/L, and in K562 cells was 26.68 μmol/L calculated by SPSS software. Overexpression or knockdown of AC026150.8 has no effect on cell activity, but affects the drug sensitivity of cell to Arc‐C in both KG‐1 and K562 cells (Figure 8). In KG‐1 cells, when compared with NC + Ara‐C group, cell inhibition rate in over‐expression AC026150.8 + Ara‐C group was significantly reduced after treating with Ara‐C at IC50 (*p* < 0.05) (Figure 8A). But this difference was not shown in K562 cells (Figure 8C). In both KG‐1 and K562 cells, when compared to NC + Ara‐C group, cell inhibition rate in si‐AC026150.8 + Ara‐C group was significantly increased after treating with Ara‐C at IC50 (*p* < 0.05) (Figure 8B,D).

### 3.6 AC026150.8 interacts with alternative splicing‐related proteins

**FIGURE 9:**
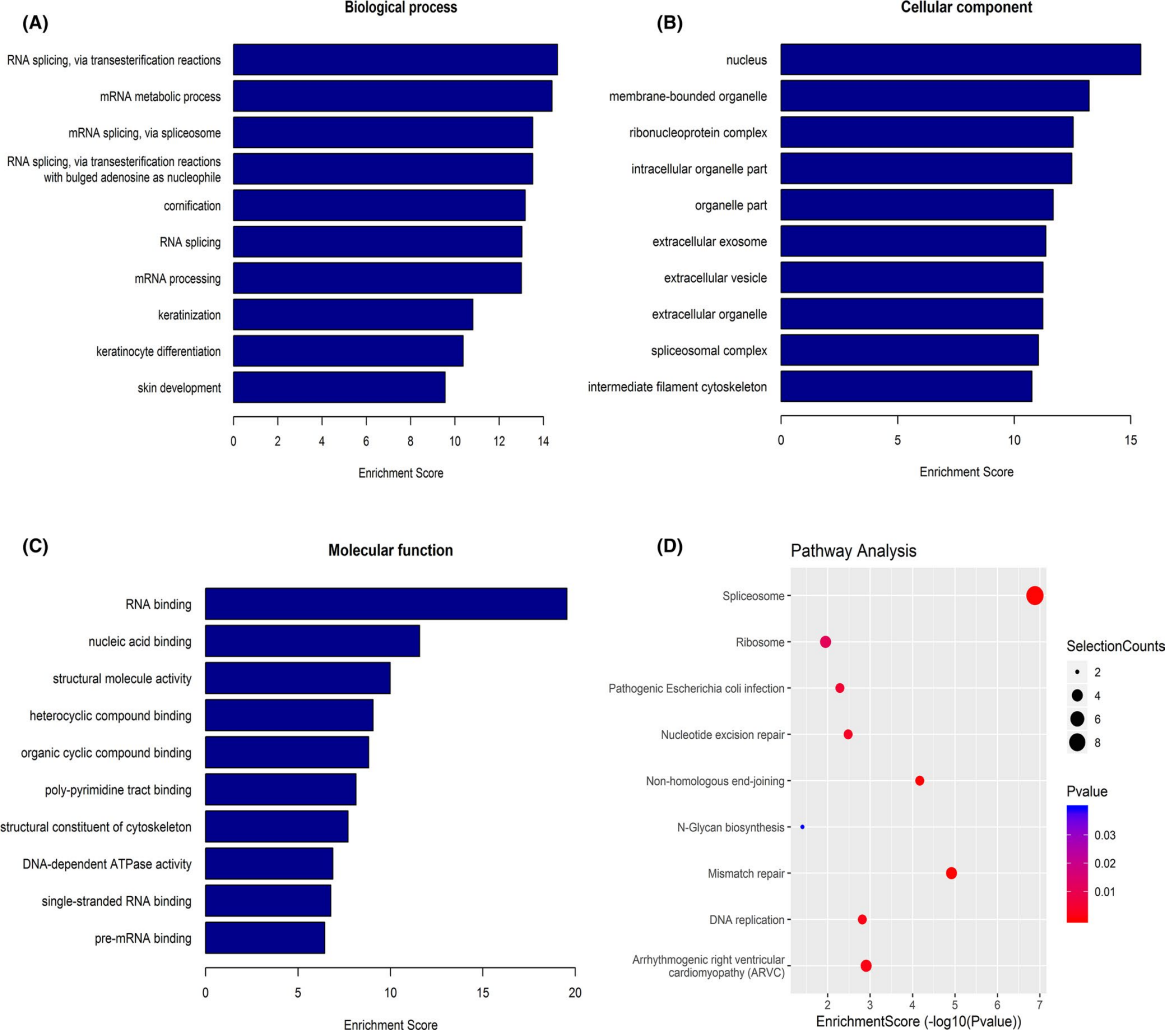
Bioinformatics analysis of the interacting protein obtained by mass spectrometry after RNA pull‐down. Gene Ontology analysis of interacting protein according to biological process (A), cellular component (B), and molecular function (C). (D) Pathway analysis of interacting protein

**FIGURE 10:**
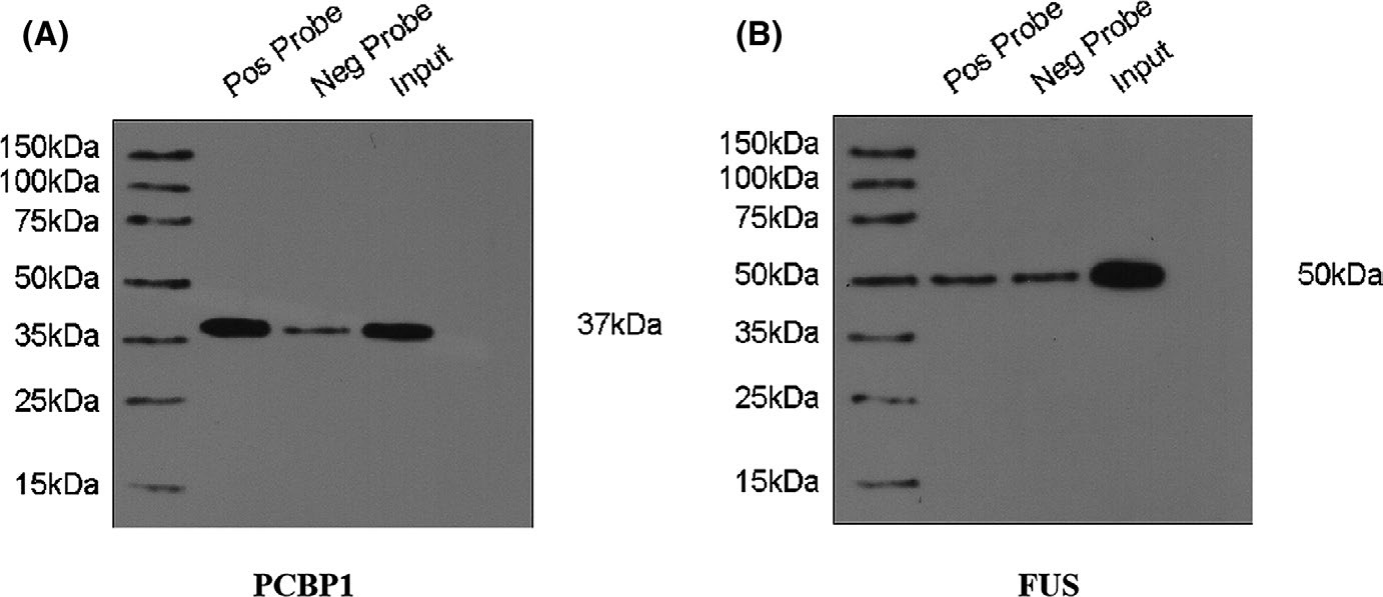
Western blot verified the combination of AC026150.8 with FUS and PCBP1. (A) AC026150.8 can bind to the PCBP1. (B) AC026150.8 cannot bind to the FUS

Diogo M Ribeiro et al. used bioinformatics methods to predict the potential molecular scaffold functions of many lncRNAs and found that AC026150.8 may have the ability of recruiting proteins as molecular scaffolds, but not experimentally verified.^20^ We predicted the proteins that may bind to AC026150.8 using catRAPID website (http://service.tartaglialab.com/page/catrapid_omics_group), and verified by RNA pull‐down experiment and mass spectrometry analysis. Eighty‐four interacting proteins of AC026150.8 were screened out by RIP assays and mass spectrometry analysis. Gene Ontology analysis were performed to explore possible relationship between biological functions and the interacting proteins of AC026150.8. Three types of sub‐analysis were included in GO analysis: biological process (BP), cellular component (CC), and molecular function (MF). According to p value, the interacting proteins of AC026150.8 were mainly enriched in RNA splicing (GO:0000375, GO:0000377, GO:0000398, GO:0008380) (Figure 9A); for the GO cellular component analysis, were mainly enriched in nucleus (GO:0005634), ribonucleoprotein complex (GO:1990904) and spliceosomal complex (GO:0005681) (Figure 9B); and for GO molecular functions analysis, were mainly enriched in RNA binding (GO:0003723), nucleic acid binding (GO:0003676) and structural molecule activity (GO:0005198) (Figure 9C). KEGG pathway analysis indicated that these interacting proteins are mostly enriched in the RNA splicing pathway (Figure 9D). Based on the website prediction results and mass spectrometry results, we selected two proteins FUS RNA Binding Protein (FUS) and splicing factor—poly(rC)‐binding protein 1 (PCBP1) for Western blot verification. The result confirmed that AC026150.8 can bind to the PCBP1 (Figure 10).

## 4 DISCUSSION

AML is a highly heterogeneous hematological malignancy maintained by long‐term abnormal proliferation of immature myeloid cells. The poor prognosis of AML patients and relapse induced by drug resistance prompted us to find novel treatments and sensitive biomarkers. LncRNA is involved in a variety of biological processes in multiple tumors, leading to metastasis and affecting prognosis of patients. With the development of next‐generation sequencing technology, it is easier to screen out dysregulated lncRNAs. LncRNAs may become a new set of potential biomarkers for diagnosis, prognosis and treatment monitoring of acute leukemia. However, only a few abnormally expressed lncRNAs have been reported to play regulatory roles in AML progression, and the mechanism of lncRNAs participating in occurrence and development of AML is still unclear.

AC026150.8 is a newly selected lncRNA from database and it has not been studied in any tumor. We found from TCGA database and Gepia online software that AC026150.8 has abnormally increased expression in AML, and the increased expression of AC026150.8 is associated with poor prognosis. Consistently with bioinformatics analysis results, we also observed that the expression of AC026150.8 was significantly higher in the bone marrow of AML patients than that in normal controls, and elevated AC026150.8 expression was associated with the OS of AML patients. Additionally, AC026150.8 expression was associated with leukocyte count, FAB classification and fusion genes. It has been generally believed that elevated leukocyte count is a poor risk factor in AML.^20^, ^21^, ^22^ Patients with high expression of AC026150.8 are usually accompanied by high white blood cell count at diagnosis, suggesting that high expression of AC026150.8 is associated with poor prognosis. Compared with patients with M1, M2, and M3, the high expression of AC026150.8 was more often observed in M4, M5 patients, which suggested that the expression of AC026150.8 might be related to abnormal development of monocytes. AML‐M4 and ‐M5 belong to acute myelomonocytic leukemia, which is a unique subtype of AML. Studies have shown that patients with M4 and M5 have poor prognosis, which is usually associated with gene rearrangements (such as MLL gene rearrangements) and gene mutations.^23^, ^24^ Our study showed that the expression of AC026150.8 in three patients with MLL gene rearrangement was high. AML patients with MLL‐AF9 have poor prognosis, implying the high expression of AC026150.8 was associated with worse prognosis. There was no difference in the expression of AC026150.8 between M3 patients and the normal controls, suggesting that AC026150.8 did not participate in the abnormal development of promyelocytic cells in M3 patients. We also found that, the high expression of AC026150.8 was more frequently observed in patients with NPM1 mutation. Boissel et al. showed that NPM1 mutation positive patients with normal karyotypes are often associated with high white blood cell counts and involvement of monocytic lineage (M4/M5),^25^ which is consistent with our results. However, NPM1 mutations usually indicate good prognosis, that is different from our results. Due to the limitation of sample size, we are temporarily unable to analyze the effects of upregulated and downregulated AC026150.8 on the prognosis of patients with NPM1 mutations. In order to further clarify whether AC026150.8 could be an indicator of prognostic stratification for patients with NPM1 mutations, larger studies are needed.

Risk stratification of AML from NCCN guidelines can help us judge the prognosis of patients and guide the treatment. However, the therapeutic effects of patients in the same risk stratification vary markedly, indicating the underlying heterogeneity within the same risk stratification group.^26^, ^27^ Our result showed that patients with high expression of AC026150.8 had a shorter OS in both favorable and intermediate risk groups, but the difference was not statistically significant. This may be caused by the small sample size after patient stratification. In spite of that, the trend of the difference is obvious and clear. This indicated that AC026150.8 may lead to worse prognosis in patients with the same risk stratification, especially in favorable and intermediate risk groups. One thing to note is that, the expression of AC026150.8 was closed in the favorable and poor risk group. We reputed one reason is that, it is not suitable to use the expression of AC026150.8 alone for indicating risk stratification, but feasible for precise stratification based on stratification. Another is that, the sample size was small, especially in the poor risk group. In the future, we will increase the sample size for further research.

Our study showed that AC026150.8 is related to the resistance of AML cells. Overexpression of AC026150.8 increased the resistance of AML cells to Ara‐C, while knocking down AC026150.8 increased the sensitive of AML cells to Ara‐C. A number of studies have shown that a variety of LncRNA can enhance the drug resistance of AML cells,^28^, ^29^ and the AC026150.8 was firstly brought into sight. Some scholars have studied the expression of gene RNA related to the metabolism and transport of cytarabine to predict the response of cytarabine in acute myeloid leukemia, but lncRNA has not been studied.^30^

For many years, the standard chemotherapy regimen for AML has always been a combination of Ara‐C and anthracyclines (the “3 + 7” regimens, 3 days of anthracyclines and 7 days of cytarabine) in the induction phase, followed by high‐dose cytarabine for consolidation phase.^31^, ^32^ Cytarabine has always been the cornerstone of the regimen throughout the treatment of AML. For the 3 + 7 regimen, the dose of anthracycline in AML has reached its plateau (60–90 mg/m^2^), the improvement of induction results depend on the dose adjustment of Ara‐C. A study has shown that high‐dose cytarabine in induction treatment produces higher remission and survival rates than standard‐dose cytarabine, especially in adult patients younger than age 46 year with acute myeloid leukemia.^33^ However, more scholars believe that high‐dose cytarabine increased treatment‐related toxicities.^34^, ^35^ In view of this, the usage of high‐dose Ara‐C for induction remains controversial. The current researches on the dosage of cytarabine for induction were based on the age stratification. Our study provided a possibility for the application of high‐dose cytarabine from a molecular point of view, that is, due to overexpression of AC026150.8 increased the resistance of AML cells to Ara‐C, we inferred that high‐dose cytarabine would be more useful for patients with high expression of AC026150.8.

Most reports on lncRNAs are describing the sponge role through combining with miRNA in Arc‐C resistance.^36^, ^37^, ^38^ However, other mechanisms of lncRNA involving in resistance have not been reported. Our research showed that AC026150.8 has a scaffolding effect, which was partially verified and supplemented the result of Diogo M Ribeiro et al. It can recruit splicing factors, and we speculate that AC026150.8 performs abnormal splicing on its target genes to make leukemia cells resistant to drugs. AC026150.8 is expected to become a new target for solving AML relapsed drug resistance, but this needs further verification in the future.

In conclusion, this analysis revealed the differentially regulated lncRNAs expression profiles in adult AML and provided a poor prognostic assessment by AC026150.8. AC026150.8 is a novel lncRNA with scaffolding function that can increase drug resistance of AML cells. This study provides further insight into the molecular aspects of AML.

## CONFLICT OF INTEREST

All authors declare that there is no conflict interest.

## ETHICS APPROVAL

This study was reviewed and approved by the Medical Ethics Committee of Shengjing Hospital of China Medical University. All the bone marrow samples used in this study were discarded clinical samples after testing, and the clinical information of patients were collected from the hospital system. Application for exemption of informed consent has been approved by the ethics committee.

## Supporting information

Supplementaryfile_S1

## Data Availability

All data generated or analyzed during this study are included in this article and its Supplementary files.

## References

[1] Myers KC , Furutani E , Weller E , et al. Clinical features and outcomes of patients with Shwachman‐Diamond syndrome and myelodysplastic syndrome or acute myeloid leukaemia: a multicentre, retrospective, cohort study. Lancet Haematol. 2020;7:e238‐e246. 31879230 10.1016/S2352-3026(19)30206-6 PMC7984274

[2] Döhner H , Weisdorf DJ , Bloomfield CD . Acute myeloid leukemia. N Engl J Med. 2015;373:1136‐1152. 26376137 10.1056/NEJMra1406184

[3] Quintana J , Advis P , Becker A , et al. Acute myelogenous leukemia in Chile PINDA protocols 87 and 92 results. Leukemia. 2005;19:2143‐2146. 16304574 10.1038/sj.leu.2403959

[4] National Cancer Center . Guideline for Chinese Cancer Registration. People’s Medical Publishing House. 2016;59‐75.

[5] Arindrarto W , Borràs DM , de Groen RAL , et al. Comprehensive diagnostics of acute myeloid leukemia by whole transcriptome RNA sequencing. Leukemia. 2021;35:47‐61. 32127641 10.1038/s41375-020-0762-8 PMC7787979

[6] Cancer Genome Atlas Research Network , Ley TJ , Miller C , Ding L , et al. Genomic and epigenomic landscapes of adult de novo acute myeloid leukemia. N Engl J Med. 2013;368:2059‐2074. 23634996 10.1056/NEJMoa1301689 PMC3767041

[7] Slack FJ , Chinnaiyan AM . The role of non‐coding RNAs in oncology. Cell. 2019;179:1033‐1055. 31730848 10.1016/j.cell.2019.10.017 PMC7347159

[8] Bhan A , Soleimani M , Mandal SS . Long noncoding RNA and cancer: a new paradigm. Cancer Res. 2017;77:3965‐3981. 28701486 10.1158/0008-5472.CAN-16-2634 PMC8330958

[9] Fu X , Ravindranath L , Tran N , Petrovics G , Srivastava S . Regulation of apoptosis by a prostate‐specific and prostate cancer‐associated noncoding gene, PCGEM1. DNA Cell Biol. 2006;25:135‐141. 16569192 10.1089/dna.2006.25.135

[10] Arun G , Diermeier S , Akerman M , et al. Differentiation of mammary tumors and reduction in metastasis upon Malat1 lncRNA loss. Genes Dev. 2016;30:34‐51. 26701265 10.1101/gad.270959.115 PMC4701977

[11] Kopp F , Mendell JT . Functional classification and experimental dissection of long noncoding RNAs. Cell. 2018;172:393‐407. 29373828 10.1016/j.cell.2018.01.011 PMC5978744

[12] Good MC , Zalatan JG , Lim WA . Scaffold proteins: hubs for controlling the flow of cellular information. Science. 2011;332:680‐686. 21551057 10.1126/science.1198701 PMC3117218

[13] Chujo T , Yamazaki T , Hirose T . Architectural RNAs (arcRNAs): a class of long noncoding RNAs that function as the scaffold of nuclear bodies. Biochim Biophys Acta. 2016;1859:139‐146. 26021608 10.1016/j.bbagrm.2015.05.007

[14] Geisler S , Coller J . RNA in unexpected places: long non‐coding RNA functions in diverse cellular contexts. Nat Rev Mol Cell Biol. 2013;14:699‐712. 24105322 10.1038/nrm3679 PMC4852478

[15] Chen Q , Cai J , Wang Q , et al. Long noncoding RNA NEAT1, regulated by the EGFR pathway, contributes to glioblastoma progression through the WNT/β‐catenin pathway by scaffolding EZH2. Clin Cancer Res. 2018;24:684‐695. 29138341 10.1158/1078-0432.CCR-17-0605

[16] Ma M , Zhang Y , Weng M , et al. lncRNA GCAWKR promotes gastric cancer development by scaffolding the chromatin modification factors WDR5 and KAT2A. Mol Ther. 2018;26:2658‐2668. 30274785 10.1016/j.ymthe.2018.09.002 PMC6225079

[17] Liu Z , Chen Z , Fan R , et al. Over‐expressed long noncoding RNA HOXA11‐AS promotes cell cycle progression and metastasis in gastric cancer. Mol Cancer. 2017;16:82. 28441948 10.1186/s12943-017-0651-6 PMC5405470

[18] Livak KJ , Schmittgen TD . Analysis of relative gene expression data using real‐time quantitative PCR and the 2(‐delta delta C(T)) method. Methods. 2001;25:402‐408. 11846609 10.1006/meth.2001.1262

[19] Agostini F , Zanzoni A , Klus P , Marchese D , Cirillo D , Tartaglia GG . CatRAPID omics: a web server for large‐scale prediction of protein–RNA interactions. Bioinformatics. 2013;29:2928‐2930. 23975767 10.1093/bioinformatics/btt495 PMC3810848

[20] Ribeiro DM , Zanzoni A , Cipriano A , et al. Protein complex scaffolding predicted as a prevalent function of long non‐coding RNAs. Nucleic Acids Res. 2018;46:917‐928. 29165713 10.1093/nar/gkx1169 PMC5778612

[21] Greenwood MJ , Seftel MD , Richardson C , et al. Leukocyte count as a predictor of death during remission induction in acute myeloid leukemia. Leuk Lymphoma. 2006;47:1245‐1252. 16923553 10.1080/10428190600572673

[22] Tien FM , Hou HA , Tsai CH , et al. Hyperleukocytosis is associated with distinct genetic alterations and is an independent poor‐risk factor in de novo acute myeloid leukemia patients. Eur J Haematol. 2018;101:86‐94. 29624746 10.1111/ejh.13073

[23] Tsai CH , Hou HA , Tang JL , et al. Genetic alterations and their clinical implications in older patients with acute myeloid leukemia. Leukemia. 2016;30:1485‐1492. 27055875 10.1038/leu.2016.65

[24] Sano H , Shimada A , Taki T , et al. RAS mutations are frequent in FAB type M4 and M5 of acute myeloid leukemia, and related to late relapse: a study of the Japanese Childhood AML Cooperative Study Group. Int J Hematol. 2012;95:509‐515. 22407852 10.1007/s12185-012-1033-x

[25] Cheng Z , Hu K , Tian L , et al. Clinical and biological implications of mutational spectrum in acute myeloid leukemia of FAB subtypes M4 and M5. Cancer Gene Ther. 2018;25:77‐83. 29491461 10.1038/s41417-018-0013-6

[26] Xue H , Gao H , Xia H , et al. IncRNA MVIH correlates with disease features, predicts treatment response and survival in pediatric acute myeloid leukemia. J Clin Lab Anal. 2021;35(4):e23739. 33704838 10.1002/jcla.23739 PMC8059728

[27] Nie Y , Su L , Li W , Gao S . Novel insights of acute myeloid leukemia with CEBPA deregulation: Heterogeneity dissection and re‐stratification. Crit Rev Oncol Hematol. 2021;163:103379. 34087345 10.1016/j.critrevonc.2021.103379

[28] Boissel N , Renneville A , Biggio V , et al. Prevalence, clinical profile, and prognosis of NPM mutations in AML with normal karyotype. Blood. 2005;106:3618‐3620. 16046528 10.1182/blood-2005-05-2174

[29] Sun H , Sun Y , Chen Q , Xu Z . LncRNA KCNQ1OT1 contributes to the progression and chemoresistance in acute myeloid leukemia by modulating Tspan3 through suppressing miR‐193a‐3p. Life Sci. 2020;241:117161. 31837329 10.1016/j.lfs.2019.117161

[30] Yu Y , Kou D , Liu B , et al. LncRNA MEG3 contributes to drug resistance in acute myeloid leukemia by positively regulating ALG9 through sponging miR‐155. Int J Lab Hematol. 2020;42:464‐472. 32359033 10.1111/ijlh.13225

[31] Kantarjian H , Kadia T , DiNardo C , et al. Acute myeloid leukemia: current progress and future directions. Blood Cancer J. 2021;11(2):41. 33619261 10.1038/s41408-021-00425-3 PMC7900255

[32] Mayer RJ , Davis RB , Schiffer CA , et al. Intensive postremission chemotherapy in adults with acute myeloid leukemia. Cancer and Leukemia Group B. N Engl J Med. 1994;331(14):896‐903. 8078551 10.1056/NEJM199410063311402

[33] Willemze R , Suciu S , Meloni G , et al. High‐dose cytarabine in induction treatment improves the outcome of adult patients younger than age 46 years with acute myeloid leukemia: results of the EORTC‐GIMEMA AML‐12 trial. J Clin Oncol. 2014;32(3):219‐228. 24297940 10.1200/JCO.2013.51.8571

[34] Weick JK , Kopecky KJ , Appelbaum FR , et al. A randomized investigation of high‐dose versus standard‐dose cytosine arabinoside with daunorubicin in patients with previously untreated acute myeloid leukemia: a Southwest Oncology Group study. Blood. 1996;88(8):2841‐2851. 8874180

[35] Löwenberg B , Pabst T , Vellenga E , et al. Cytarabine dose for acute myeloid leukemia. N Engl J Med. 2011;364(11):1027‐1036. 21410371 10.1056/NEJMoa1010222

[36] Abraham A , Varatharajan S , Karathedath S , et al. RNA expression of genes involved in cytarabine metabolism and transport predicts cytarabine response in acute myeloid leukemia. Pharmacogenomics. 2015;16:877‐890. 26083014 10.2217/pgs.15.44 PMC7115907

[37] Zhang H , Liu L , Chen L , Liu H , Ren S , Tao Y . Long noncoding RNA DANCR confers cytarabine resistance in acute myeloid leukemia by activating autophagy via the miR‐874‐3P/ATG16L1 axis. Mol Oncol. 2021;15:1203‐1216. 33638615 10.1002/1878-0261.12661 PMC8024725

[38] Hu N , Chen L , Wang C , Zhao H . MALAT1 knockdown inhibits proliferation and enhances cytarabine chemosensitivity by upregulating miR‐96 in acute myeloid leukemia cells. Biomed Pharmacother. 2019;112:108720. 30970520 10.1016/j.biopha.2019.108720

